# Morpho‐functional variation and response pattern of microglia through rodent ontogeny showing infant microglia as stable and adaptive than matured

**DOI:** 10.1002/brb3.2315

**Published:** 2021-08-06

**Authors:** Anirban Ghosh, Payel Ghosh, Ishani Deb, Sandip Bandyopadhyay

**Affiliations:** ^1^ Immunobiology Laboratory Department of Zoology Panihati Mahavidyalaya Kolkata West Bengal India; ^2^ Department of Zoology School of Sciences Netaji Subhas Open University Kolkata West Bengal India; ^3^ Department of Biochemistry University of Calcutta Kolkata West Bengal India; ^4^ Department of Biochemistry KPC Medical College and Hospital Kolkata West Bengal India

**Keywords:** cytokine, development, microglia, ontogeny, perinatal, primary culture

## Abstract

Microglia, myelo‐monocytic lineage cells, that enter in the developing brain at early embryonic stages and integrate in CNS, are involved in almost all neuroinflammatory conditions. We studied how microglia change their responses through the development and maturation of brain in normal physiological conditions using an ex situ model to delineate their age‐specific morpho‐functional responsiveness. Rapidly isolated microglia from different age‐matched rats were characterized with Iba1^+^/CD11b/c^+^/MHCclassII^+^, cultured, studied for cell‐cycle/proliferative potency, ROS generation and phagocytosis, viability and morphological analysis induced with GMCSF, MCSF, IL‐4, IL‐6, IL‐10, and IFN‐γ. The study showed marked differences in cellular properties, stability, and viability of microglia through ontogeny with specific patterns in their studied functions which were coherent with their in situ morpho‐functional attributes. Phagocytic behavior showed a notable shift from ROS independence to dependence toward maturation. Perinatal microglia were found persistent in ex situ environment and neonatal microglia qualified as the most potent and versatile responders for morpho‐functional variations under cytokine induced conditions. The study identified that microglia from infants were the most stable, adaptive, and better responders, which can perform as an ex situ model system to study microglial biology.

## Graphical abstract

Microglia, myelo‐monocytic lineage cells involved in almost all neuroinflammatory conditions were studied for their response through the development and maturation of brain in normal physiological conditions using an ex situ model for investigating their age‐specific morpho‐functional responsiveness. The study showed marked differences in stability and viability of microglia through ontogeny with specific patterns in their proliferation, ROS dependent phagocytosis and cytokine responsiveness through maturation stages. Microglia from infants were found stable, adaptive and better responsive than matured, which can perform as an ex situ model system to study microglial biology.

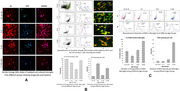

## 1 INTRODUCTION

Microglia are the tiny glial component of CNS tissue serving as sentinels in the delicacy of brain and found active in almost all neuroinflammatory situations. Their roles in neuronal aging, pathogenesis, degeneration, trauma, stroke, or psychiatric ailments have established them as the major immune‐competent cells in brain capable of scrutinizing brain microenvironment for subtle changes and maintaining CNS health (Aloisi, 2001; Nimmerjahn et al., 2005; Paolicelli et al., 2011; Prinz & Priller, 2014). They are the unusual candidates in brain who integrate in CNS originating from hemopoietin progenitors with myelo‐monocytic lineage specificity (Prinz & Priller, 2014; Rezaie & Male, 2002). The controversial issue of microglial origin in brain has not ended yet as some favor early entry of yolk‐sac macrophages into the presumptive embryonic brain; others report the entry and colonization of monocytic cells in perinatal or early postnatal brains through peripheral routes (Ghosh et al., 2015; Ginhoux et al., 2013; Lassmann et al., 1993; Rezaie & Male, 2002). Therefore, microglia occupy a unique position with versatile functions, primarily for defending the CNS tissue, but capable of exerting a reverse double‐edged effect in the brain compartment (Rivest, 2009; Santiago et al., 2017).

Plenty of works are now revealing the variety of microglial functions related to different neurological disorders and other adversities. The changes in morpho‐functional aspects from development to growth and maturation and aging have drawn attention till date, and our understanding evolved from the traditional ramified and amoeboid concepts to contemporary resting/surveillent functional reality of microglia in living tissue (Del Rio‐Hortega, 1932; Nimmerjahn et al., 2005; Streit et al., 1999; Wake et al., 2009). With the emerging opinion on developmental plasticity and cellular heterogeneity or adaptability of the so‐called microglia or brain antigen presenting cells or monocyte progenitors of brain (Ghosh, 2010; Santambrogio et al., 2001), it is now assumed that insight to the changes in microglial behavior in a normal growth continuum or ontogeny may possess the key to the core understanding of microglial response in various maladies in brain from diseases to aging.

Therefore, postulating that microglia change with the maturation continuum, we tried to develop an ex situ model system to work with microglia from different developmental and maturation age groups by adopting a rapid isolation protocol for the cells from the rodent brain. Analyzing some basic cellular properties for maturation, morphological response, stability, and adaptability or variability against an array of cytokines were documented to determine the best responding microglial age group. This was a challenging attempt to assess a delicate cell like microglia ex situ from different age groups and stabilize them in culture for further experimentation. This may provide us valuable insights of microglial biology in a normally developing and maturing brain, and offers an effective model system to work with microglia in a similar platform for more intricate aspects of its response behavior in a developing age continuum or ontogeny.

## 2 MATERIALS AND METHODS

### 2.1 Animals and grouping

The female Sprague‐Dawley rats were maintained for the experiment, and were fed with hind liver pellets or equivalent and water ad libitum; 12 h light and dark cycles were maintained, examined, and weighed at regular intervals throughout the experimental period. Reproductively matured male and receptive females were set for breeding at a rate of 1:2, respectively, examined for copulation plug and other symptoms for mating to separate pregnant mothers and pregnancy days were counted to obtain the required embryos. Neonates were maintained with their respective mothers in one cage as they were at waning age. The groups of animals maintained were—a) Late embryo (ED 18 ± 2), b) neonate (D 5 ± 1), c) young adult (D 45 ± 5), and d) mature adult (D 240 ± 10), where ED = embryonic days (calculated from the day of mating/copulation) and D = days after birth. For late embryos, the pool of all embryos from each pregnant mother and for neonates, the pool of all pups from each waning mother, and each individual from the other two groups had been considered as *n* = 1. In each experimental setup, *n* = 5 repeats were done. Animals were not sexed for the late embryonic (ED 16–18), neonate (D5), and young adult (D45) groups as it was not validly possible and for matured adult (D240 ± 10) the results of two males and three females have been accumulated to form a data set for statistical interpretation, hence neutralizing the sex effect.

### 2.2 Rapid isolation of microglia from different age groups

The rats were deeply anaesthetized with sodium pentobarbital (50 mg/kg body weight) and transcardially perfused with ice‐cold PBS to ensure the removal of monocytic cells present in blood. Whole brain was dissected out and placed into ice‐cold PBS containing 1% penicillin‐streptomycin solution (P/S), mechanically dissociated and enzymatically digested for 30 min at 37°C by 5–15 U type II collagenase (Sigma‐Aldrich) and 500 U DNase I (Sigma‐Aldrich). Cell suspensions in ice‐cold PBS passed through a cell strainer (BD Falcon, USA) of porosity ∼70 μm for perinatal brains and ∼100 μm for adults to make a single cell suspension, allowed to adhere on a glass petri dish (DURAN, Czech Republic) for an hour in 5% CO_2_ humified environment at 37°C (CO_2_ Incubator Galaxy 48S, New Brunswick, Germany). The adherent cells were then recovered with 1X Trypsin‐EDTA (Sigma‐Aldrich) solution followed by addition of DMEM (Gibco, Life Technologies, USA) with 10% FBS (Gibco, Life technologies, USA). Recovered cells were resuspended in media, laid on 20%/70% Percoll (GE Life Sciences, USA) gradient and centrifuged at 2500 rpm for 25 min at 18°C (Thermo Scientific‐HERAEUS Megafuge 8R, USA). Collected cells with highly enriched microglia from the interface were suspended in DMEM containing 1% P/S antibiotic antimycotic solution (Himedia, India) along with 10% FBS. Their viability and density were immediately measured by the trypan blue exclusion method in Neubauer improved chamber (Marienfeld, Germany) and observed under Microscope Nikon Eclipse TS 100 (Nikon Corporation, Japan). In the case of embryos and neonates, the whole brain of 5–6 pups or embryos were pooled together and treated with half‐dilution of enzyme concentration for the initial steps of brain tissue digestion in contrast to adult rats and with some minor modifications in cell strainer and centrifugation.

### 2.3 Ultra‐microscopy of isolated cells (scanning electron microscopy)

The cells immediately isolated from brain tissue were fixed in 2.5% glutaraldehyde (Sigma‐Aldrich) for 4 h at 4°C followed by washing in PBS. The sample was gradually dehydrated in graded alcohol and finally brought to 100% acetone followed by critical point drying, spread on a 1 cm^2^ grease‐free glass slide placed on a metallic stub with conducting silver paint. The cells laid on the glass platforms were then coated with gold‐palladium alloy of 100–200Å thickness in a diode sputtering system. Finally, the samples were observed in FEI Quanta‐200‐MK2 (30KV), USA scanning electron microscope.

### 2.4 Immunophenotyping of isolated microglia from different age groups

The cells were suspended in PBS (1–2 × 10^6^ cells/ml), mildly fixed with 2% paraformaldehyde, washed in PBS, and stained with antibodies as selected. Before staining, cells were blocked in 5% FBS in PBS (blocking buffer) for 1 h to avoid nonspecific binding. The cells were then washed, initially tagged and incubated with anti‐Iba1 mAb (Abcam) for 1 h, washed and incubated with fluorescenated 2° Ab in the dark for 1 h. After washing, the cells was again incubated with fluorescence conjugated CD11b/c mAb (Biolegend) in the dark for 1 h, washed, recovered, and prepared for flow cytometric analysis, assessed in BD FACSVerse flow cytometer, USA in blue laser (wave length 488 nm) excitation in FITC channel for Alexa Flour 488 conjugated CD11b/c and in PE channel for PE/Texas Red conjugated Iba‐1 and reading were analyzed and represented with BD FACSuit software for immunophenotyping of the cells.

### 2.5 Approaches to establish microglia primary culture from different age groups of brain

Immediately isolated cells from different age groups were prepared and attempted for primary culture. Initially, the cells were counted for assessing viability and density by trypan blue and morphological variations among different groups were observed and documented by Microscope Nikon Eclipse‐TS‐100, using CCD Camera (DS‐Fi2‐U3) and NIS Elements BR software (Nikon Corporation, Japan). As per cell count data, the isolated cell density was adjusted to seed for culture at a concentration of 1 × 10^6^ cells/ml and cells were plated in 35 mm, 6 well and 12 well cell culture plates (BD Falcon Multiwell, USA) to establish primary culture for short and long term. Using both RPMI and DMEM media, finally it was standardized to be maintained in DMEM containing high glucose (d‐glucose), l‐glutamine + 15% FBS + 2% P/S. The cultures were placed in a 5% CO_2_ humidified closed environment at 37°C in CO_2_ Incubator. The media of the culture were routinely changed at 3 days interval and scheduled to maintain for 2 weeks and more for all groups. The cellular morphology, viability, and immunophenotyping were scheduled to assess with the interval of 3 days, 6 days, and so on to check the culture conditions and to decide the continuity of culture.

### 2.6 Development of variable in vitro cytokine environment for microglia of different age groups, viability, and morphometric assessment

After attempting to establish the primary culture for each group, to create an in vitro reductionist differentiation environment, we charged the selected cytokines separately in specific doses with days’ interval with five different cytokines important for monocytic cell lineage differentiation. The cytokines applied were recombinant GMCSF, MCSF, IL‐4, IL‐6, and IFNγ procured from PROSPEC, Israel. Isolated cells from each group were seeded in duplicate for each cytokine in triplicate sets of experiments separately in the 5% CO_2_ humified environment at 37°C. After 24 h of seeding of cells at an average conc. 1 × 10^6^ cells, they were charged with cytokines (MCSF, GMCSF, IL‐4, IL‐6, and IFN‐γ) at variable concentration of 1, 3, 9, 50, and 100 ng/ml in 96‐well plate for MTT assay for checking cell viability and at the variable concentration of 1, 3, and 9 ng/ml in 35 mm, 12 well or 24 well cell culture plates for cell culture for morphometric analysis and incubated in the same CO_2_ humified environment with induced cytokines.

The viability of microglia of different groups in different cytokine conditions were tested by the standard MTT assay. Briefly, microglial cells were cultured in 96 well plates, mostly for 24 h after cytokine induction, cells were washed and incubated with MTT (Himedia, India) solution added with a final concentration of 0.5 mg/ml in PBS dissolved with DMSO (SRL, India)/MTT solubilization buffer in the culture plate and then was scanned in the Multi‐well Plate Reader (Epoch Microplate Spectrophotometer, BioTek, USA) for measuring the optical density at 570 nm wavelength of light.

The cells in culture were analyzed for their morphometric parameters to see the effect of these cytokines and cell shape determination. Their responses to each cytokine separately were measured at different concentrations. This study design was aimed to select the optimal cytokine dosing as well as the maximal responsive or sensitive microglial age group that could be handled in in vitro conditions to develop a model culture system. In these sets of experiments three different variables, that is, cytokines (MCSF, GMCSF, IL‐4, IL‐6, and IFN‐γ), concentration of cytokines (1, 3, 9, and 50 ng/ml) and different developing/maturation ages of microglia (ED18, D5, D45, and D240) were taken into account where experiments for each age groups were done at least thrice in duplicate sets. The cellular adaptive morphology of microglia from different developmental age groups in culture with trigger of different cytokines was separately measured under bright field and phase contrast microscopy and photomicrographs of the cells were taken in Nikon microscope as mentioned earlier. Three major cellular morphological parameters were considered for morphometric analysis, that is, (1) single cell area, (2) single cell length, and (3) cell projection length, and measurements were done after 48 h (2 days) in culture. After analyzing this plethora of data (see statistical analysis also), the desired age group of isolated microglia for their responsiveness and optimal cytokine concentration to work has been generalized.

### 2.7 Immunostaining of isolated and cultured microglia from different age groups

Immunophenotyping of cells had been done with CD11b/c (Biolegend) and Iba1(Abcam), for their acceptability as microglial markers and for culture additionally MHC class II (Biolegend) and Acridin Orange (AO) (Sigma‐Aldrich) were also included counterstained with DAPI (Himedia, India). Fixing with 2–4% paraformaldehyde, they were washed and permeabilized by 0.2% Triton X‐100 and aspirating Triton, the cells were rinsed in PBS followed by blocking in 5% FBS in PBS (blocking buffer) for 1 h. After washing differently conditioned cells in culture plates were incubated with diluted anti‐Iba1 mAb for1 h, washed, and incubated with fluorescenated 2° Ab (PE/Texas Red, Abcam) in dark for 1 h. The cells were again incubated with fluorescence conjugated CD11b/c in dark for 1 h, washed and then DAPI was used in PBS dilution of 1:1000 in dark and incubated for staining the nuclear DNA. Finally, the cells were washed and prepared for microscopic observation. For staining with MHC class II (Alexa Fluor 647) the same procedures were maintained. For AO staining, this method has been described in the following Section 2.9. Then the plates were viewed through Nikon trinocular inverted microscope with epifluorescence attachment (Eclipse TS 100‐F, Nikon Corporation, Japan) using Epi‐FL Filter Block MA‐FL‐C‐DAPI Filter (Nikon, Japan) for DAPI staining, Epi‐FL Filter Block N B‐2A (Nikon, Japan) for FITC/Alexa Fluor 488 and Epi‐FL Filter Block N G‐2A (Nikon, Japan) for PE/Texas Red/Alexa Fluor 647 conjugates. Photographs were taken using CCD camera (DS‐Fi2‐U3, Nikon, Japan), processed, and analyzed with NIS Element BR Software (Nikon, Japan) for studying the immunophenotype of differently conditioned cells.

### 2.8 Analysis of cell cycle and proliferative capacity of microglia at different age groups

Cell cycle phases of the microglial cells were analyzed with propidium iodide staining and measured by flow cytometry. Initially, cells were trypsinized and harvested, then cells were fixed with 70% Ethanol and incubated overnight at 4°C. After removing ethanol and washing with PBS, the cells were treated with RNase A (Purelink RNaseA, Invitrogen) for 1–2 × 10^6^ cells/ml and incubated for 1 h at 37°C. Then the cells were added with 20 μl of propidiumiodode (PI) (Invitrogen) from the stock (1 mg/ml) and incubated for 15 min at 37°C. The cells were assessed in BD FACSVerse flow cytometer, USA, in blue laser (wave length 488 nm) excitation in PE channel for Propidium Iodide and readings were analyzed.

Proliferative capacity of microglia was measured by Ki67, a nuclear antigen associated with cell proliferation. For fluorescence microscopy, cultured cells were fixed in 4% paraformaldehyde, washed, and permeabilized using permeabilisation buffer (PBS + 5% FBS + 0.25% Triton X‐100). Then cells were incubated with diluted FITC conjugated Ki67 antibody (Novus Biologicals) for 1 h in dark, visualized under a fluorescence microscope using Epi‐FL filter Block N B‐2A and documented, processed and analyzed with NIS Element‐BR Software. The process of Ki67 staining is the same up to RNase A treatment for flow cytometry. The cells were then permeabilized with 0.25% Triton‐X and 1% FBS dissolved in PBS and washed, resuspended cells were incubated with 6–8 μl of diluted FITC conjugated Ki‐67 and incubated at 4°C and then washed in PBS before flow cytometric analysis. Then, the cells were assessed in BD FACSVerse flow cytometer, USA, in blue laser (wave length 488 nm) excitation in FITC channel using Alexa Fluor 488 conjugated Ki67 and readings were analyzed. Both the readings of PI and Ki67 were represented with BD FACSuit software for measuring nuclear proliferation.

### 2.9 Assessment of phagocytic potential of microglia from different age groups

The phagocytic potential of microglia in cultured condition after isolated from different age groups were measured by AO both by fluorescence microscopy and flow cytometry. Initially from the stock solution of 1 mg/ml of AO (Sigma‐Aldrich) the working solution of 2.5 μg/ml in PBS was applied to the cells and kept in 5% CO_2_ environment at 37°C followed by removal of excess AO. Then the plates were viewed under the microscope by using either Epi‐FL Filter Block N B‐2A or Epi‐FL Filter Block N G‐2A (Nikon, Japan) as AO staining is visible in both the filters in green and red channels for nucleus and lysosomal vesicles respectively, photographed, processed, and analyzed with NIS Element BR Software (Nikon, Japan).

For flow cytometric quantification of AO uptake, after preparing the single cell suspension, as stated earlier, they were treated with AO at a conc. of 2.5 μg/ml to the cells (1–2 × 10^6^ cells/ml) suspension and incubated at room temperature in dark, washed to remove excess AO and resuspended for acquisition in flow cytometer. The cells were assessed in BD FACSVerse flow cytometer, USA with blue laser (wave length 488 nm) excitation in PerCP‐Cy 5.5 channel for AO and readings were analyzed and represented with BD FACSuit software.

### 2.10 Assessment of reactive oxygen species in microglia from different age groups

The reduced non‐fluorescent H_2_DCFDA can be oxidized and converted into fluorescent 2′, 7′‐dichlorofluorescein (DCF) by intracellular ROS. In this protocol, we applied H_2_DCFDA to label the intracellular ROS and detected the DCF intensity by flow cytometry. Cells were harvested by trypsinization, centrifuged, and washed with PBS and dissolved the pellet in PBS at a density of 1–2 × 10^6^ cells/ml and then DCFDA (Sigma‐Aldrich) was added at the final concentration of 20 μM. The cells were then incubated in dark and prepared for measuring the fluorescence in flow cytometer. The resultant fluorescence intensity was measured with control in BD FACSVerse flow cytometer, USA with blue laser (wave length 488 nm) excitation in FITC channel for resulting DCF fluorescence, and readings were analyzed and represented with BD FACSuit software.

### 2.11 Immunohistochemistry of formalin‐fixed‐paraffin‐embedded rat brain tissue sections

Deeply anesthetized (sodium pentobarbital, 50 mg/kg body wt.) rats intracardially perfused with 4% paraformaldehyde in 0.1 M PBS and with 5% sucrose were dissected out for brain and the tissue samples were post‐fixed in same solution and stored. After routine histological preparation and sectioning of brain tissue cortex with 15 μm thickness were preheated overnight, xylene washed, gradually rehydrated, washed in PBS and mildly fixed in 2% paraformaldehyde, and washed. Blocking was done with 5% FBS in humified chamber at ambient temperature, washed and incubated with 1° antibody overnight (1:700 dilution), that is, anti‐Iba1 and anti‐MHC class II (Abcam), and Alexa‐Fluor 488 conjugated anti‐CD11b (Biolegend), then washed and incubated for 2° antibody, that is, PE/Texas Red conjugated goat antibody (Abcam) (1:1000 dilution) for an hour and washed. For double staining, same processes were repeated and finally nuclear staining were done with DAPI (Abcam), semidried and mounted in DPX and observed under Eclips TS 100‐F epi‐fluorescence microscope using DS‐Fi2‐U3 CCD camera and NIS‐BR software (Nikon Corporation, Japan) with fluorescent filters as described in sub‐section 2.7.

Silver staining‐gold toning were employed to detect microglia in situ (McCarter, 1940). Slides with tissue sections were treated with ammoniacal aqueous solution after initial deparaffinization and passed through 10% Globus’ hydrobromic acid solution. Washed slides were put into 50% aqueous silver carbonate, passed through 5% formalin, washed and placed in 1% aquous gold chloride solution, fixed in 5% sodium thiosulfate solution, washed and dehydrated in graded ethanol, finally mounted in DPX and observed under the same microscopic system in bright‐field and documented as mentioned earlier.

### 2.12 Organotypic brain slicing and in situ observation of microglia with NDPase staining

After deep anesthesia as mentioned above the rats were intracardially perfused with 5% sucrose in PBS and brains were removed, cut into small squares and placed to filter papers with super‐glue gel properly using tweezers and immediately submersed in PBS‐sucrose solution. Brain pieces of cortex region attached with vibratome platform by glue‐gel were coronally sliced in Leica VT 1200S vibratome for 200 μm sections, collected and introduced in 0.1 M PBS with 7.5% sucrose at 7.4 pH (PBSS) and immediately processed for NDPase histochemistry (Almolda et al., 2013). The brain slices were replaced in 0.1 M TMB and pre‐heated at 37°C. Incubation medium was prepared with substrate Inosine‐5′ di‐phosphate, Sigma in TBM, added with manganese chloride and lead nitrate solution and filtered. Organotypic slices were placed in incubation medium at 37°C for 30 min with shaking, removed, washed in PBSS, exposed to ammonium sulphide solution briefly, washed and stabilized by brief use of 1% silver nitrate solution, washed and mounted in gelatin‐coated slides, dried, and counter‐stained with 0.1% toluidine blue. Then sections were dehydrated in graded ethanol, exposed in 1‐butanol for 1 min and submerged in xylene, mounted DPX, and observed under Nikon TS 100‐F, and documented as mentioned earlier.

### 2.13 Statistical analysis

All the assays were performed at least thrice or more for each age group using isolated microglia in culture and in duplicate sets or as specifically mentioned in the methods. The collected data were analyzed statistically with mean values and standard deviations for cellular functional parameters. The results of MTT assay were calculated with mean values and standard deviations, statistical differences were analyzed by one way repeated measure ANOVA and pair‐wise multiple comparisons by Tukey post‐hoc test. The large data sets generated from cellular morphometry for highest cell area, cell length, and/or projection length were analyzed and represented in the box plot with median values and deviation ranges through the Sigma Plot V13.0 software. For best responding group selection, all differences in the median values of age group had been tested for significance at α level 0.05 represented in box plots with median values and deviation ranges with a multiple comparison test by Tukey's method.

## 3 RESULTS

### 3.1 Rapid isolation of microglia from developmental/maturation stages and characterization

**FIGURE 1:**
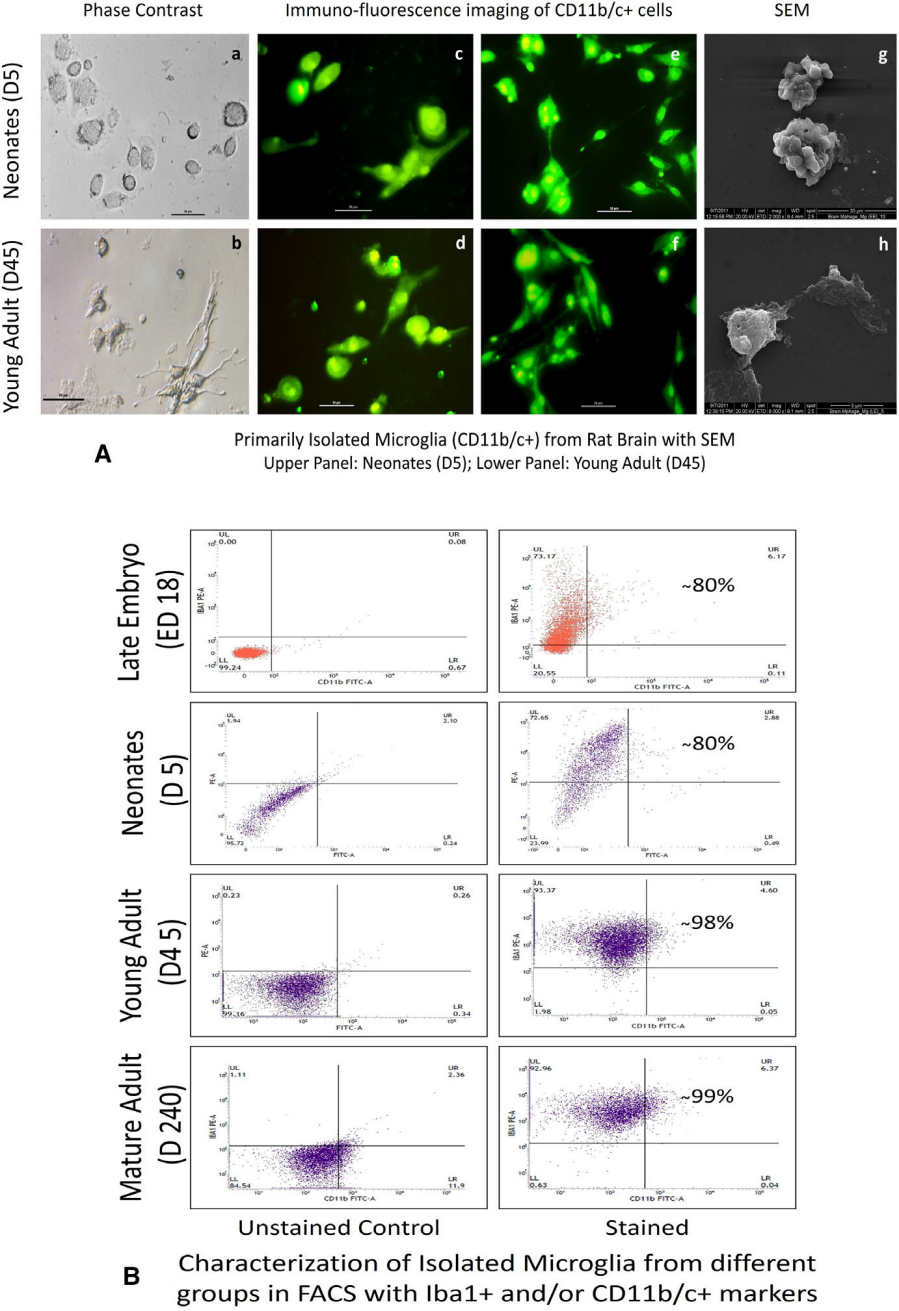
Characterization of microglia isolated from different age groups. (A) shows morphological variation of microglia isolated from neonates/pups and young adult by phase‐contrast microscopy followed by immunofluorescence for CD11b/c and SEM study. Upper row: Microglia from neonates—(a) mostly spherical with short projections in few cells, (c,e) expressing CD11b/c with round morphology with few bipolar, elongated structure, (g) ruffled surface membrane observed in SEM. Lower row: Microglia from young adults—(b) extended morphology of cell along with spines bearing surfaces, (d,f) CD11b/c^+^ microglia showing ramified structures and (h) in SEM microglia showing smooth surface with thread like projections from body and elongated appearance. In (B), flow cytometric analysis for Iba‐1^+^ and CD11b/c^+^ cells showing representative dot‐plot analysis obtained after gating unstained control in first column. Microglial was characterized on Iba‐1^+^/CD11b/c^+^ markers with percentage of purified cell (cumulative value for either or both marker positive cells) showing in second column of each group

Microglial cells were isolated from four selected age groups with the modified protocol as mentioned earlier. Mostly, comparatively smaller cells with rounded morphology were isolated from the late embryonic and neonatal rat brain, whereas cells with elongated morphology were found isolated from adult brains (details of structural variations are described in Figure legend) (Figure 1). After isolation, the cells were also characterized with microglia/macrophage lineage marker anti‐Iba‐1 (intracellular/cytoplasmic localization calcium binding protein) antibody and anti‐CD11b/c (membrane bound monocytic cell lineage marker) antibody simultaneously to find the % positive cells by flow cytometric analysis, which demonstrated that the isolation procedure reliably yielded highly purified/enriched microglia (∼80% population in case of late embryo and neonates whereas in adults it reaches to > 98%) population. This characterization showed purity of isolated cells in different groups, particularly adults showing highest Iba1^+^ and CD11b/c^+^ cell population (Figure 1).

### 3.2 Establishment of microglia primary culture through developmental/maturation stages and immunophenotyping with lineage and antigen presenting markers

#### 3.2.1 Establishment of primary culture

**FIGURE 2:**
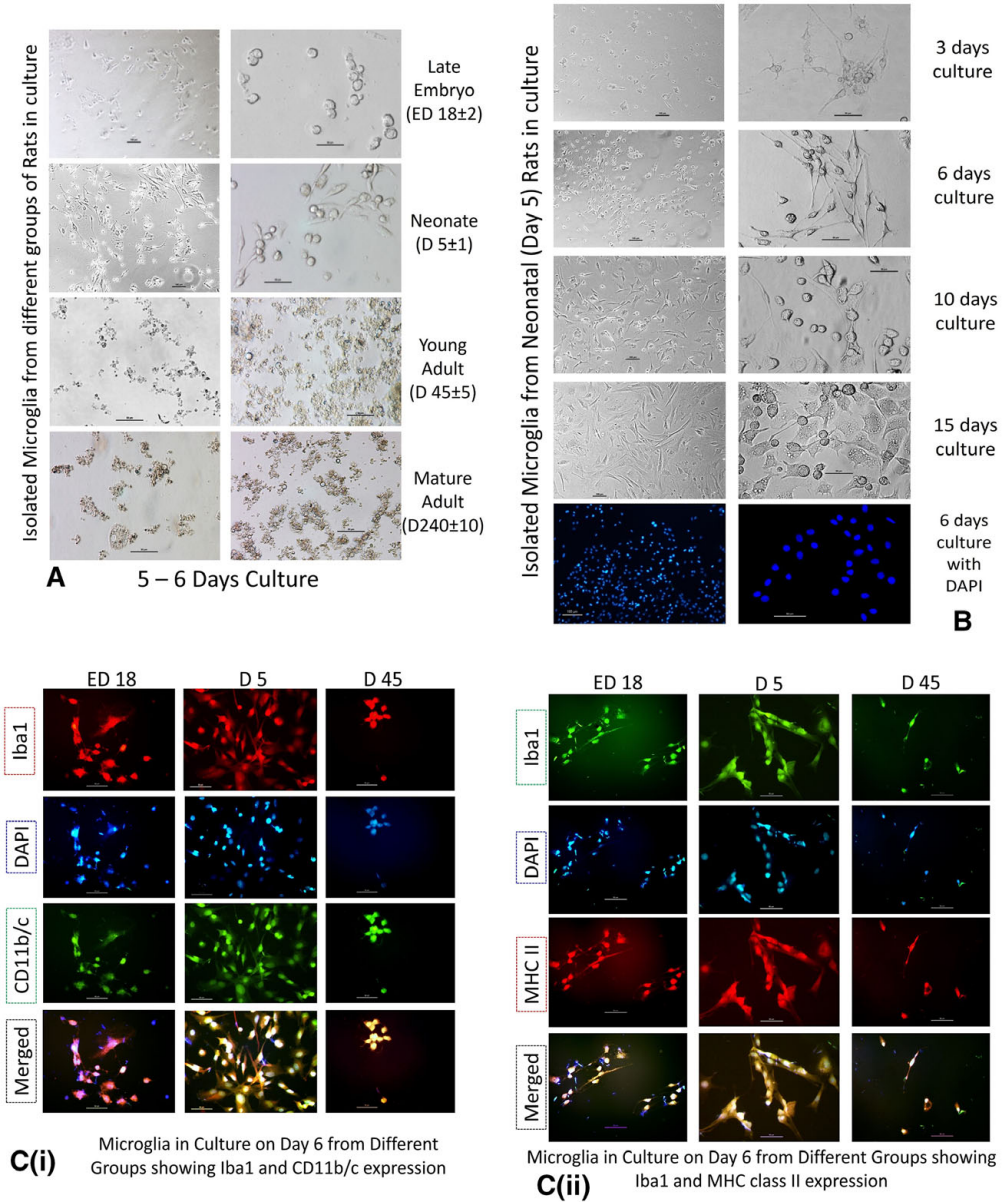
Establishment of primary culture of microglia, receptor expression and characterization. Isolated microglia in culture for 5–6 days from different age groups shows healthy cell populations from perinatal groups (ED18 ± 2 and D5 ± 1) in the (A). The cells are showing moderate confluence and mostly rounded with few elongated morphology in culture. Elongation increases in neonatal cells than late embryonic cell isolates in culture with higher confluency. In contrary, unhealthy cells with granularity and apoptotic features are found after 5 days from adult groups (D45 ± 5 and D240 ± 10) while maintaining the same conditions. In (B), microglial cells isolated from neonatal rats kept in culture for 3, 6, 10, and 15 days in vitro (DIV), immunostained for their nuclei with DAPI (blue, in case of 6 day culture) and their morphology were analyzed. Phase contrast microscopy images at 3 DIV show microglia of mostly amoeboid with ovoid shape with only a few cells show ramified bipolar morphology. At 6 DIV, microglia cells become more homogeneous with more cells presenting highly branched processes forming ramified morphology. At 10 DIV, cells exhibit distinct monolayer of highly differentiated microglia with dense branching and at 15 DIV, showing enlargement of highly proliferating microglia cells extending their protrusions occupying whole surface. Microglial cell's nuclei are shown with DAPI (blue) cultured for 6 day (100X and 400X). In (C), cells stained with fluorescenated antibody counterstained with DAPI (blue) shows their retention of microglial lineage features in vitro as all group microglia were either Iba1+ and CD11b/c+ or Iba1+ and MHC class II+ in both of the blocks of the figure, where infant microglia showed stable population and dual positivity with extended bipolar morphology with much higher confluency; but in D45 culture cells showing less amount of cells with dual positivity and nearly no cells were found for mature adults 6^th^ day in vitro to characterize. (Scale bar = 50 μm)

One of the primary challenges of this work was to establish the primary culture of microglia from different age groups. After modification and standardization, we found the more healthy response of cell populations isolated from late embryo and neonatal rat brains in culture in contrast to cells from young adults and matured adults. Isolated cells from both late embryos (ED 18 ± 2) and neonates (D 5 ± 1) showed over 80–90% viability in trypan blue exclusion method with healthy morphology and higher confluency at 5–6 days in vitro (DIV). But cells cultured from adults showed viability of 30–20% or worse with unhealthy cells and granularity for the same period in vitro (Figure 2A). Microglia from late embryos and neonates can continue their healthy morphology up to 3 weeks and more with repeated media changes in every 72 h. However, after 2 weeks they started to show changes in morphological features and adherence properties (Figure 2B).

#### 3.2.2 Characterization of primary culture of microglia

After establishing primary cultures from different groups, the receptor expression profiles of those cells were verified in 5–6 DIV to check the microglial and mocnoytic/macrophage lineage properties with CD11b/c and Iba1 counter stained with DAPI. As adult cells tend to discontinue at 5–6 DIV, the poor number of cells with CD11b/c^+^ co‐expressed with Iba1^+^ has been documented for young adults only. In contrary, cultured cells expressed core signature markers CD11b/c and intracellular Iba1 which are found to be expressed in almost entire population in microglia isolated and cultured from neonatal and late embryonic rats after 5–6 DIV. To observe that whether microglia continue to hold their functional attribute of antigen presentation in vitro, MHC class II expression of them from different groups in 5–6 DIV had been assessed with Iba1 expression as microglial lineage identity and counterstained with DAPI. Almost every cell was found to express MHC class II as found in the merged microphotograph for microglia with similar poor cell numbers in adults (Figure 2C).

### 3.3 Assessment of cell cycle and proliferation of microglia ex situ through developmental/maturation continuum

**FIGURE 3:**
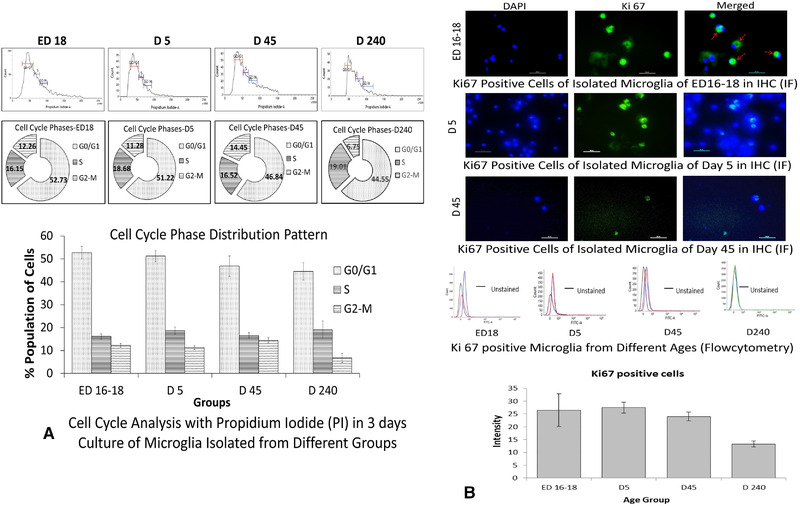
Assessment of cell cycle phase distribution pattern and proliferative capacity. (A) Microglia cells isolated from late embryos, neonates, young adults, and matured adults in short term culture were assessed for cell cycle phase distribution among groups stained with propidium iodide (PI) and subjected to flow cytometric analysis. With representative histogram plots in FACS, the percentages of cells in each phase of cell cycle are represented in doughnut plot and bar diagram. (B) In the next three panels the microphotographs are showing the Ki67 localization (indicated by red arrows) and expression (green) on the microglial cells in culture from different age groups counterstained with DAPI (blue). Next panel shows the representative FACS histograms of Ki67 expression in different groups and following bar diagram is showing the % positive Ki67 populations for each groups of microglia in culture. Both bar diagrams are expressed as mean ± SD, *n* = 3. No significant differences found in cell cycle phases between ED16‐18, D5, and D45, but significant differences found for G2‐M phase in D240‐270 compared to others in (A), whereas similar differences found for Ki67 expression in D240–270 (*p* ≤ .05)

Based on propidium iodide staining, DNA content in different phases of cell cycle in the isolated microglial cell population were determined for different groups in culture. The observed results are documented in Table 1, which showed that the S phase populations of cells ranged within 15–20% within groups while the successive G2‐M phase cells ranged between 11% and 15% for first three age groups indicating effective conversion to the next cell cycle. However, a remarkable decrease occurred in G2‐M phase distribution in matured adults, showing ∼7% of microglia in that age group (Figure 3A). When microglia from different groups in culture were compared for their Ki67 expression in immunofluorescence microscopy counterstained with DAPI, it was observed that a particular population among them were Ki67^+^ and their intensity showed a decrease in postnatal and adult groups than late‐embryonic group. When % of Ki67^+^ cells in each group were quantified in flow cytometry, it was found that the mean values for perinatal and young adult microglia expressing Ki67 in culture were within 25–27% of the total population. However, a remarkable half‐fold decrease occurred for matured adult microglia, restricting it around 13% of the population. This half‐fold decrease of the proliferating marker Ki67 in matured adult microglia was found corroborated with the decrease of G2‐M microglia found in the cell cycle phase distribution studies with PI and meant the downfall of the proliferating potential of microglia with maturation and aging (Figure 3B).

**TABLE 1:** Cell cycle phase distribution among isolated microglia from different age groups

**Group**	**G0/G1**	**S**	**G2‐M**
**ED 16–18**	52.73% ± 2.79	16.15% ± 1.12	12.26% ± 0.81
**D 5**	51.22% ± 2.45	18.68% ± 1.51	11.28% ± 0.81
**D 45**	46.84% ± 4.48	16.52% ± 1.34	14.45% ± 1.12
**D 240**	44.55% ± 3.81	19.01% ± 3.96	6.75% ± 2.09

### 3.4 Evaluation of phagocytic potential and reactive oxygen species production of microglia ex situ through developmental/maturation continuum

#### 3.4.1 Phagocytic potential

**FIGURE 4:**
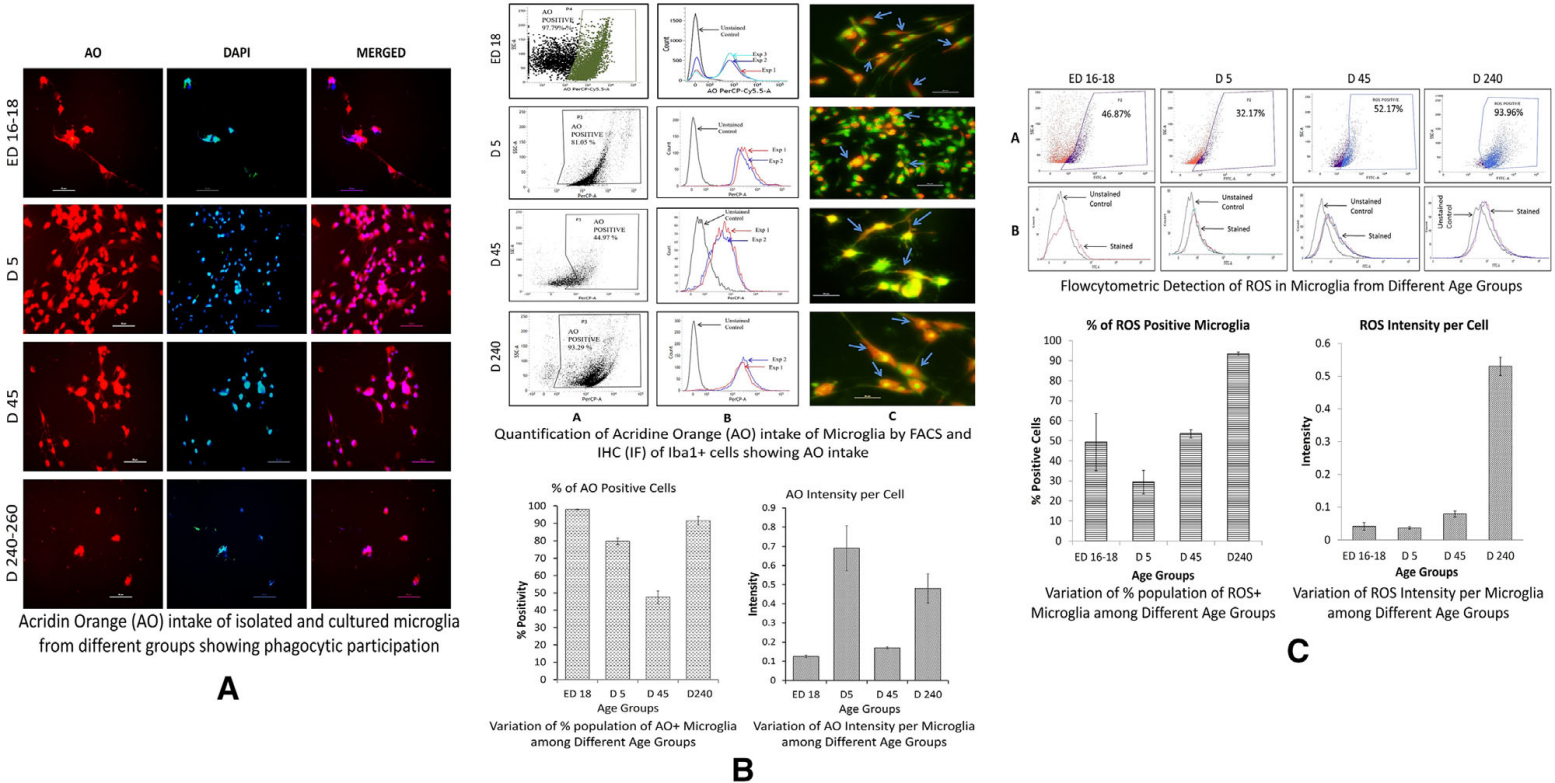
Evaluation of phagocytic potentiality and cellular ROS generation. In (A), immunocytochemistry for AO (red), nuclear counterstain with DAPI (blue) is showing that mostly the cells of different groups overlap major portions of their cell body with DAPI and AO positivity. Overall, the property of microglia as basic phagocytic cells is also seen by using the auto‐exposure mode of microphotography (Scale bar = 50 μm). (B) is showing flow cytometric estimation of microglia cells engulfing acridine orange (AO) in first two rows and immunofluorescence photomicrographs of cultured Iba1^+^ microglia with phagocytosed AO in third row in controlled exposure mode. Percentage of AO positive cells are shown for each group in 1^st^ row, histograms of fluorescence intensity of AO engulfing cells against unstained control showing shift of peaks due to AO uptake in 2^nd^ row, followed by Iba1^+^AO^+^ staining in 3^rd^ row (engulfed acridine orange particles were indicated by blue arrows) (Scale bar = 50 μm). Variation in % population of AO positive cells in FACS is represented graphically in lower half of 2^nd^ block and significant differences observed among groups (*p* ≤ .05). In (C) the results are represented in bar diagrams for both % positivity of microglia for ROS and relative intensity of DCF depicting ROS intensity per cells across the prenatal to matured age groups where the upper two panels (A and B) are showing the representative FACS data for each groups with % positivity and intensity profile respectively. Significant differences observed among groups (*p* ≤ .05) expect ROS production intensity per microglia within ED16‐18, D5, and D45 groups

Acridine Orange (AO), a cationic molecule which can stain phagolysosomes emitting orange spectra and bind to nucleic acids as well, has been used for documenting cells with phagocytic properties through immune‐fluorescence (IF) microscopy and in flow‐cytometric analysis (FACS), respectively. In an auto‐exposure capture mode in CCD camera, the fluorescence spectra showed almost every cell in culture with varied confluency in different groups were positive for AO in a red filter counterstained by DAPI (Figure 4A) expressing the phagocytic nature of microglia in general. Phagocytosis were assessed in 3 DIV microglia among different groups for comparison. Fluorescence microscopy showed that during postnatal period, activated (also called amoeboid) microglia display very intense granular red, yellow, or orange staining. In flow cytometry, red shift demonstrated higher AO positive populations where over 95% cells were AO^+^ in both neonates and matured adults, but young adults showed around 45% positivity (Figure 4B). In photomicrographs, it was seen that all Iba1^+^ cells had phagocytic capacity to AO uptaken in their cytoplasm in young and particularly in mature adults. However, microglial cells from neonates were not showing such global AO uptake by Iba1^+^ cells like mature adults, rather showing highly AO containing cytoplasm where found. In contrast, microglia from young adult showed that ramified microglia had uptaken AO through their projection as bead‐like structures and showed fewer amounts of lysosomes than activated microglia. It also showed some cells with marginal AO or no AO in their cytoplasm, thus suggesting that resting or surveillant microglia may not contain as many lysosomes as the activated forms. In case of matured/aged rat, red spectrum emission was observed throughout the cytoplasm and almost all cells were AO^+^ in their cytoplasm in accordance with a very high reading for AO in FACS and highly corroborative with the FACS data represented graphically in Figure 4B.

#### 3.4.2 ROS production

It was found that when the ROS positive microglia from late embryonic brain showed a mean of ∼50%, the mean of neonatal ROS producing microglial population decreased to ∼30% or one‐third of the population in vitro. However, the ROS positive cells again increased to about half of the population and that increase continued to reach a high peak in the matured adult where almost the whole population of microglia were capable of producing ROS (∼95%) as shown in 3^rd^ block of Figure 4. Interestingly, ROS generation per cell in terms of DCF conversion from H_2_DCFDA, showed a relative increase of ROS production with development and maturation. Late embryonic and neonatal microglia were showing minimal capacity of mean ROS production per cell, but it increased two folds in young adults from perinatal microglia. However, a remarkable hike in ROS generation potential occurred in cells which were isolated from matured aging brain and the observed increase was about 10 folds in comparison to perinatal microglia and about 5 folds from young adult microglia (Figure 4C).

### 3.5 In situ morpho‐functional attributes of microglia through developmental and maturing stages

#### 3.5.1 Iba1^±^CD11b/c^±^ or MHC class II^±^ microglia in situ

**FIGURE 5:**
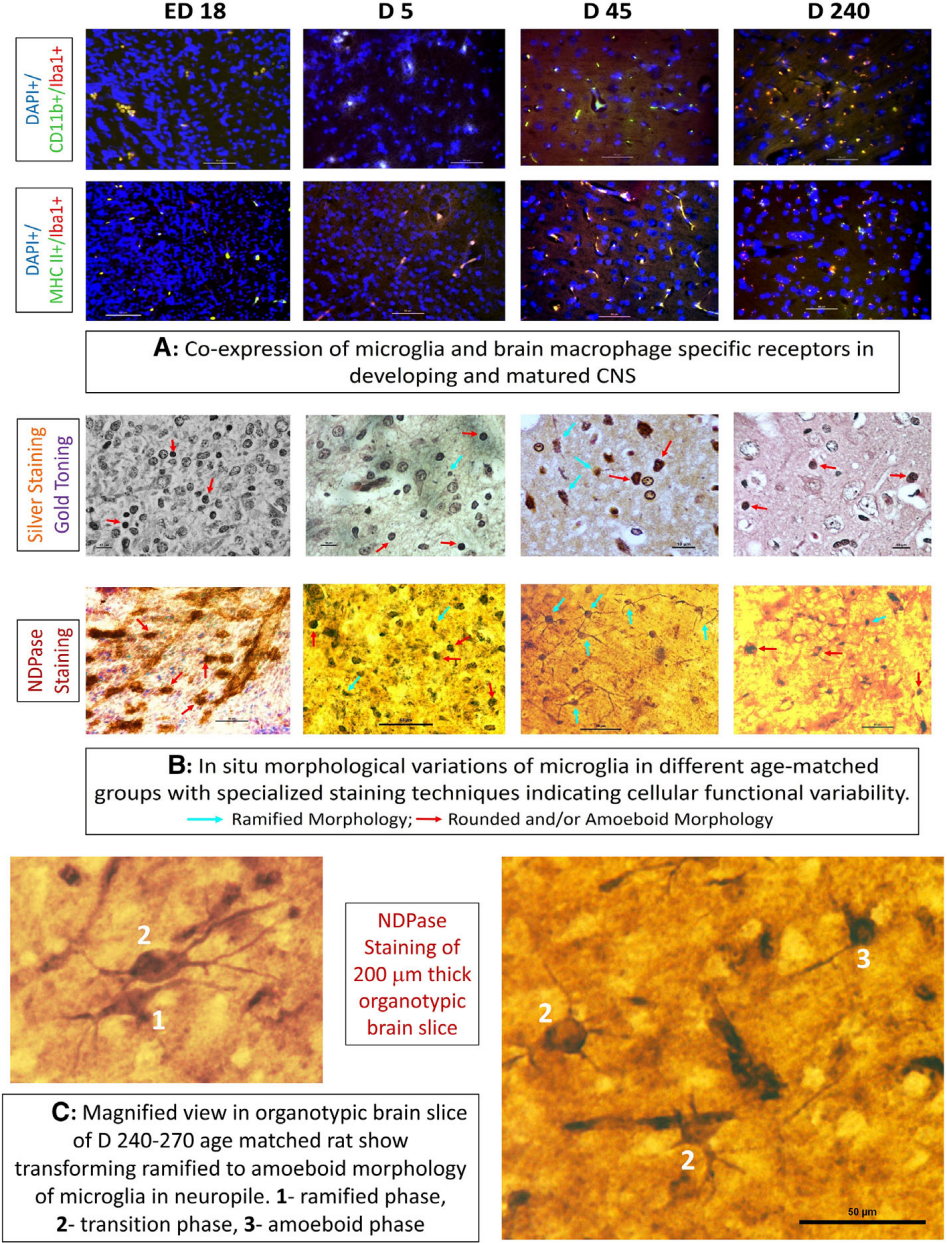
In situ morpho‐functional attributes of microglia with development and maturity. In (A) distribution of microglia in brain cortex of different age matched groups, viz., late embryo (ED16–18), neonates (D5), young adult (D45), and mature adult (D240–270) have been detected in FFPE sections using microglia specific markers Iba1+ and CD11b/c+ in the first panel and in the second panel antigen presenting properties of distributed microglia in brain have been shown with Iba1+ and MHCclassII+ specific markers (Scale bar = 50 μm). In (B), distribution and morphological variations of microglia have been shown in different maturation states in first panel using silver‐gold staining technique where rounded/amoeboid and ramified forms are indicated with red and blue arrows respectively (Scale bar = 10 μm). In second panel, microglial forms and ramifications are clearly visible and indicated separately by NDPase staining in organotypic brain slice. Presence of amoeboid cells in ED16‐18 and D5 group transforms to ramified forms in D45 and redifferentiated to amoeboid shape in D240‐270 (Scale bar = 50 μm). In (C), transformation of ramified microglia to amoeboid form in organotypic brain slice in the matured adult (D240–270) stage has been shown through steps 1 → 3 using NDPase staining (see text for details)

Immunohistochemical staining for microglia with cell specific markers showed the presence of microglia in the cortex region of the brain of different maturation ages. It has been observed that for late embryo Iba1^+^CD11b/c^+^ microglia are arrayed in tandem in many places of developing brain cortex where Iba1^+^MHCclassII^+^ microglia are dispersed. Presence of Iba1^+^CD11b/c^+^ and Iba1^+^MHCclassII^+^ cells increased in mature adult stage where higher expression of MHCclassII+ indicated toward increased antigen presenting function of microglia during mature adult stages (Figure 5A).

#### 3.5.2 In situ morphological variations

Specialized staining technique with silver impregnation and gold toning in brain tissue classically applied to identify microglia in brain sections indicated on its distribution and morpho‐patterning with ages. It is evident from microphotographs that their shapes were more rounded and condensed in late embryonic (ED16‐18) and neonatal brains (D5) which transformed with branching projections in young adults (D45), whereas, considerable proportions of such cells showed an amoeboid morphology in mature adults (D240–270). This patterning was more prominently deciphered when NDPase reactivity staining was applied for observing microglia in different developmental/maturating stages (Figure 5B). Rounded dense cellular cytoplasm of late embryonic (ED16–18) and neonatal (D5) stages transformed to highly ramified forms with projecting filapodia or fine branching surveilling brain parenchyma were observed in organotypic brain slices in the young adults (D45). Whereas, both ramified and amoeboid morphs of microglia were observed in mature adult brain slice with distinctive cytoplasmic functional activation with NDPase action (Figure 5B).

#### 3.5.3 Reactive microgliosis

The microglial morpho‐functional existence of transitional forms and their co‐existence in neighboring positions were clearly identified in mature adult (D240–270) stage. Organotypic sections showed the intermediate stage when a ‘ramified’ microglia (1) was transforming into a more rounded intermediate stage (2) and then to an irregular bulging shaped ‘amoeboid’ reactive microglia (3) in a stepwise manner (Figure 5C). Such phenomena, classically termed as ‘reactive microgliosis’ had been documented in mature adult representing their reactive state in those age‐matched brain. Such microgliosis, which more frequently observed in pathogenic brain tissue, was also documented in the normal aging brain indicating toward the presence of more active state of microglia, hence supporting our ex situ findings of phagocytic potential, ROS production, and related instability in primary culture.

### 3.6 Effect of different cytokines on microglia ex situ at different maturation stages on their viability and morphology

#### 3.6.1 MTT assay for viability

**FIGURE 6:**
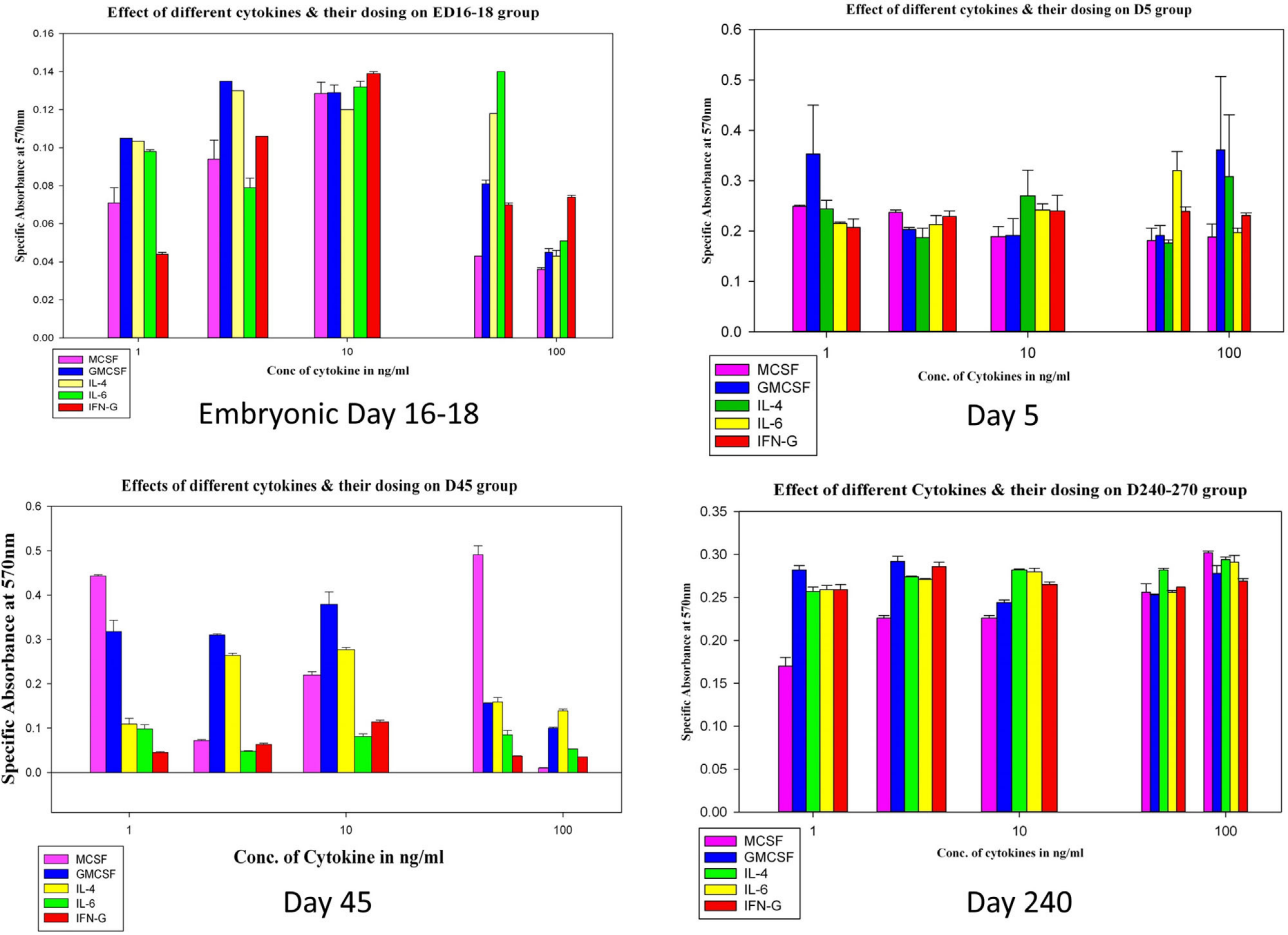
MTT assay for detection of cellular viability of isolated microglia with different cytokines with variable concentrations. MTT assay to measure viability of microglia in culture in conditioned media by selected cytokines MCSF, GMCSF, IL‐4, IL‐6, and IFNγ respectively as shown in graph legends with variable concentrations from 1 to 100 ng/ml for each developing/maturing age groups where X axis designates cytokine concentrations in log scale and Y axis designates absorbance reading. The box plots are derived in Sigma Plot 13.0 software. Results differ among groups, cytokines and dosing (*p* ≤ .05), where ED16‐18 group shows high viability, D5 group shows moderate viability and variability when exposed to different cytokines and dosing, D45 group shows low viability and high variability, and D240‐270 group shows lowest viability and less variability. For details see text

The results when compared, it was found that the viability of cells from the prenatal embryonic days was less (ranging within 0.04–0.13) compared to the other age groups. For neonates, the viability range was in between 0.2 and 0.4 in different cytokine dosing environments, but for young adults variation of viability of cells in response to variable cytokines and dosing was wider (0.04–0.5) showing over 10 folds *in group* variability differences where other groups showed a maximum of 2–3 folds *in group* variation in viability. Lower viability range (0.17–0.3) and minimal viability variations were found in the matured adult groups initially (after 24 h of seeding), although they were unable to continue in culture for longer. Thus, within low viability ranges in general, microglia isolated from neonatal brain had shown better survival with a lower fluctuation range (within two folds) and had the potential to continue for a longer period in culture as previously seen (Figure 6).

#### 3.6.2 Choice of best dosing of selected cytokines

The best dosing selection for each cytokine for each group had been selected in short‐term culture where the best responses were documented as the highest cell area, cell length, and/or projection length. The results of different cytokine dosing responses on different cellular morphological parameters in ex situ microglia isolated from different age groups had been represented in Figures in Supporting Information. The findings and the choice of the best dosing option for different cytokines are summarized in Table 2.

**TABLE 2:** Best responsive doses of cytokines for isolated microglia from different age groups

**Cytokines**	**Parameter**	**ED 16–18**	**D5**	**D45**	**D240–270**
**MCSF**	Area	1 ng/500 μl	3 ng/500 μl	9 ng/500 μl	1 ng/500 μl
	Body length	1 ng/500 μl	9 ng/500 μl	9 ng/500 μl	1 ng/500 μl
	Projection length	1 ng/500 μl	9 ng/500 μl	9 ng/500 μl	1 ng/500 μl
**GMCSF**	Area	1 ng/500 μl	1 ng/500 μl	9 ng/500 μl	1 ng/500 μl
	Body length	1 ng/500 μl	3 ng/500 μl	1 ng/500 μl	1 ng/500 μl
	Projection length	1 ng/500 μl	3 ng/500 μl	1 ng/500 μl	1 ng/500 μl
**IL‐4**	Area	1 ng/500 μl	9 ng/500 μl	9 ng/500 μl	3 ng/500 μl
	Body length	9 ng/500 μl	3 ng/500 μl	3 ng/500 μl	3 ng/500 μl
	Projection length	1 ng/500 μl	1 ng/500 μl	9 ng/500 μl	3 ng/500 μl
**IL‐6**	Area	9 ng/500 μl	1 ng/500 μl	3 ng/500 μl	9 ng/500 μl
	Body length	9 ng/500 μl	3 ng/500 μl	3 ng/500 μl	9 ng/500 μl
	Projection length	9 ng/500 μl	9 ng/500 μl	3 ng/500 μl	9 ng/500 μl
**IFN‐γ**	Area	1 ng/500 μl	3 ng/500 μl	3 ng/500 μl	1 ng/500 μl
	Body length	3 ng/500 μl	9 ng/500 μl	1 ng/500 μl	1 ng/500 μl
	Projection length	3 ng/500 μl	9 ng/500 μl	3 ng/500 μl	1 ng/500 μl

### 3.7 Selection of best responding microglial group in reductionist cytokine environment

**FIGURE 7:**
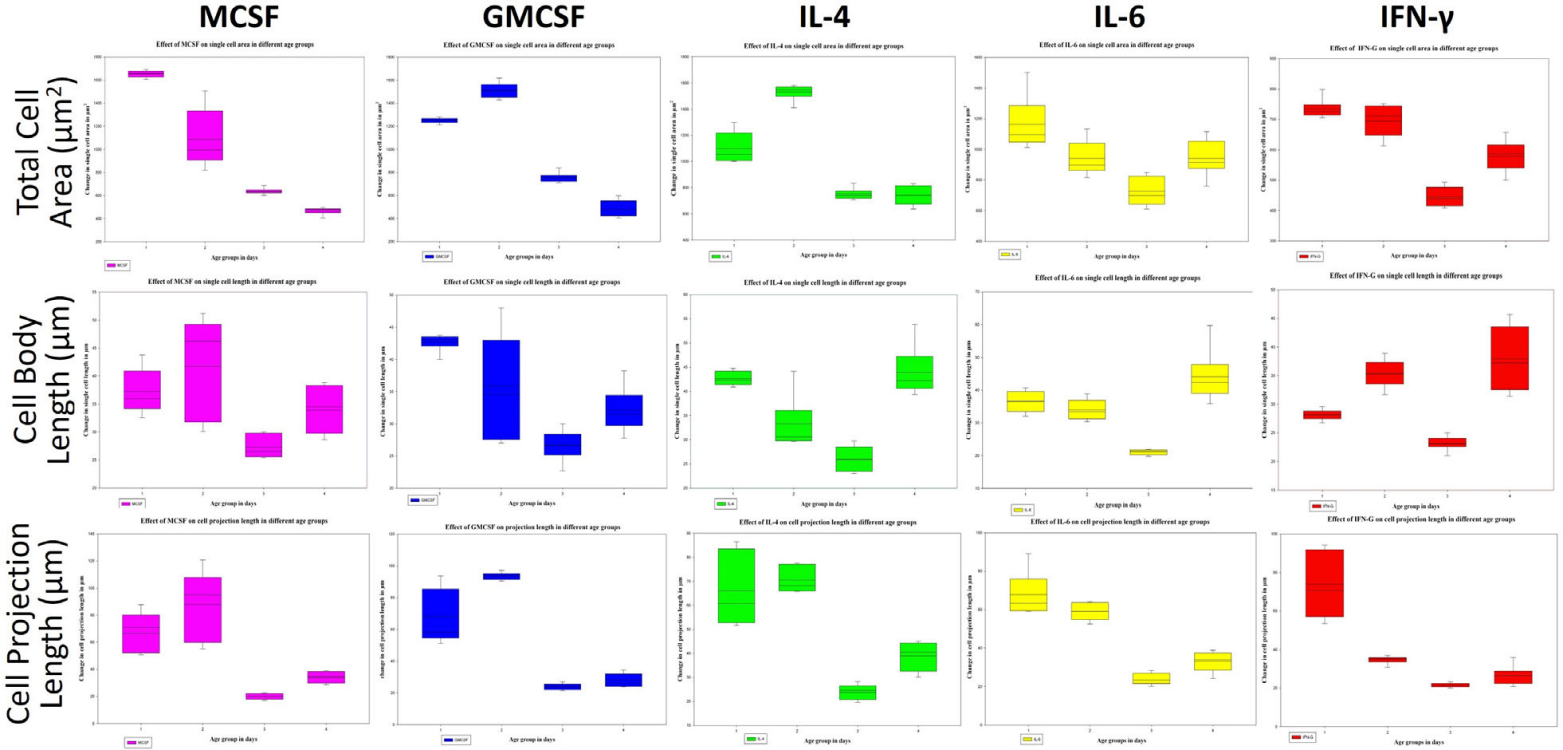
Selection of best dosing response against selected cytokines in group of microglia. In the figure, microglial morphological parameters with best dosing responses as selected from previous section (Figures in Supporting Information) are represented in the box plots with median survival and deviations tested for significance and multi variant comparison (*p* ≤ .05). Plots are directly inserted as obtained in Sigma Plot 13.0 software. Cytokine effects only for best dose responses for each group are represented in columns and morphological parameters of microglia in rows, where X‐axis for each graph bears four age groups and Y‐axis shows parametric magnitude. Results compared in Figure 7 selected ED16–18 and D5 as better responders showing higher responses in most cases (see also Table 2 and text)

As per the best responsive doses as selected from the above data sets, the cellular morphological parameters of each developmental and maturation age group were compared to determine which group is most responsive to the selected dose. The best dosing for every cytokine for each parameter, for each group were compared further to detect which group showed the maximum changes and variability in morphometry (Figure 7). The neonates (D5) was seen as the most responsive with maximized cell area in GMCSF, IL‐4, and IFN‐γ induction but for MCSF and IL‐6 microglia from prenatal age group ED16‐18 showed maximum expansion in area. However, in most cases, the variability of changes among ED16‐18 and D5 groups were less for single cell area. For MCSF and GMCSF cytokines, D5 group showed the highest variability with maximum changes in single cell length. Cytokines IL‐4, IL‐6 and IFN‐γ showed maximum cell length in microglia in matured adults (D240–270), but less differences in variability with postnatal group. In case of cell projection length with MCSF and GMCSF cytokines, ED16–18 and D5 showed maximum changes and particularly microglia in D5 showed the highest variability in cell projection length when induced with GMCSF. Cytokines IL‐4, IL‐6, and IFN‐γ showed maximized cell projection length for microglia in ED16–18 but almost marginally differed for IL‐4 and IL‐6 in microglia at D5, except for IFN‐γ which varied considerably with maximized length for the age group ED16–18 in comparison to D5. From the above observations, we found neonatal infants and late embryos as groups which showed maximal changes in cytokine responses. However, most of the maximal changes shown by microglia in vitro were from neonatal (D5), that is, with 6 highest and 8 next highest median values out of 15 combinations (Figure 7).

## 4 DISCUSSION

With the approach to extract delicate microglia in healthy conditions from complex brain tissue in different developing/maturing phases of normal healthy rodent individuals and assessing cellular behavior and responses through ontogeny, we found specific key performance indicators for microglia. Most studies on microglia are involved with different patho‐physiological conditions including inflammation, aging, trauma, or behavioral/psychiatric disorders (Colonna & Butovsky, 2017; Dutta et al., 2016; Walker & Yirmiya, 2016). From discovery, a number of studies were also undertaken to decipher its origin and integration in brain with immune functional significance (Nayak et al., 2014; Rezaie & Male, 2002; Saijo & Glass, 2011). But, there are very few studies which address the changing morpho‐functional aspects of microglia in a developing or maturing brain from prenatal to matured stage with in situ imaging and functional studies (Czeh et al., 2011; Paolicelli & Ferretti, 2017). But in situ study of cellular behavior and properties have several limitations. Proper understanding of normal microglial behavioral modifications with maturation and aging may hold the key to understand its disease response, and we designed our study to document this. To reach this goal, a primary ex situ model platform for microglia isolated from different age groups was essentially developed as the primary challenge of the study instead of using microglia cell lines like HMO6, BV2, N9, or MG20 etc. particularly for their inability to produce results in age‐wise comparison (Iwamaru et al., 2007; Stansley et al., 2012).

There are different approaches to isolate microglia and continue with primary culture, but most have their limitations. Generally, two basic approaches are followed for microglia isolation from brain tissue. One follows the method of prolonged shaking after preparing the mixed glial culture from tissue and other utilizes the density gradient centrifugation mostly from mechanically and enzymatically dissociated tissues (Frank et al., 2006; Ghosh et al., 2007; Hassan et al., 1991; Sedgwick et al., 1991). Other approaches are selecting and sorting cells by their receptors (Garcia et al., 2014; Gordon et al., 2011). For the first one, primarily prepared mixed glial suspension needs prolonged culture and shaking and the final yield of microglia requires weeks, thus altering the cellular properties from actual in situ condition. Alternatively, the enzymatically dissociated brain preparation is subjected to density gradient centrifugation for nearly an hour, causes stress to microglia with less yield. Moreover receptor‐based sorting methods are rapid, but too restricted and dependent on expression of surface receptors that actually varies with development and age, hence causing partial recovery of microglial population in varied conditions. Keeping these issues in mind and as per the demand of the present study, we approached by utilizing cellular properties of adherence with a short enzyme treatment following density gradient centrifugation (Ghosh et al., 2007; Hassan et al., 1991; Sedgwick et al., 1991). The purpose was to get cells rapidly from tissue and generate more yield in better functional conditions. Matrix adherence is a classic property of monocyte/macrophage cells (Giulian & Baker, 1986; Ling et al., 1983; Rustenhoven et al., 2016 ) and used here for a short period in comparison to prolonged adherence and incubation of mixed glial cultures in other methods (Giulian & Baker, 1986), thus helping in shortening centrifugation time and stress. These cells when characterized, showed required microglial properties (Figure 1). Extending them in culture, healthy growth of microglia from perinatal stage was observed in contrast to poor continuation of cells in vitro from adult (Figure 2). Long term culture potential of perinatal microglia was also depicted in the study and extended beyond 3 weeks with almost cent percent confluency leading to a supra‐basal layer formation of cells with differing morphotypes (data not shown). On the other hand, although we found exceptional purity of microglia from adults by immunophenotyping with Iba1+, CD11b/c+, and MHC class II+ marker expression (≥∼98%) in isolated cells, but showed poor response in culture and unable to extend in vitro. This stressed and restricted response of matured microglia ex situ can be correlated with their cellular feebleness found in functional parameters as observed later in this study, but also partially resulted from probable cellular damage during isolation due to their vulnerable extended and ramified morphology entangling the brain tissue in situ (Nimmerjahn et al., 2005; Rezaie & Male, 2002; Streit et al., 1999). This is indicating the reason that why already established most microglial cell lines from mice, rodent or human, namely, BV2 or N9, HAPI, and HMO6, respectively, are mostly developed from perinatal/neonatal brain and do not satisfy the age‐oriented modelling of microglial function (Galatro et al., 2017; Horvath et al., 2008; Timmerman et al., 2018). This successful isolation of microglia from different age groups and establishment of primary culture for neonates with continuity fulfilled a challenging aspect of the study with further potential to work with the cellular model of microglia.

Proliferative and phagocytic potential both showed developmental and maturation age‐sensitive variations (Figures 3 and 4). In general, microglia have been reported as having restricted proliferative capacity, but may alter varying degrees in differently conditioned brains (Askew et al., 2017; Gomez‐Nicola et al., 2013; Mander et al., 2006). However, there is no specific reporting for age‐wise estimation of their proliferative capacity as we observed. Recent reporting of Askew et al., 2017 showed that microglia maintains a steady population by a spacio‐temporal coupling of self‐renewal process through a balance in between proliferation and apoptosis, and estimated this renewal process as pretty fast unlike previously thought (however much less than monocyte/macrophage) (Askew et al., 2017). Our observations showed specific trends in proliferative potential. Cells from matured adults showed a drastic reduction of G_2_‐M phase, decreasing to nearly half of the previous groups, showing ∼6–7% of microglia in G_2_‐M and similarly Ki67 showed microglial proliferation reduced half the amount in matured adults (∼13%) in comparison to other groups (∼24–27%) (Figure 3). However, the estimation of self‐renewal ranging below 1% at a given time, as reported can be justified with the estimation of apoptotic spillage at that time in the neuropil, however such self‐renewal may be faster if depleted in the natal brain (Askew et al., 2017; Huang et al., 2018). The strikingly low OD in microglia in MTT assay from the late embryonic brain indicates very less mortality and in reverse may have a chance to rapidly increase in number in the developing brains against a merely constant proliferating rate in all compared age groups. This may be due to a much higher difference between proliferation and mortality when average proliferation rates are not changing much within groups (Figures 3 and 6). Therefore, the prominent trend of decreased proliferating capacity with maturity leading to aging should be noted as an important performance indicator of the cell when considering the microglial physiology in any condition from maturity to aging. This observation can be correlated with another observation where alternatively activated microglia of M2 phenotype show retracted renewal capability and that might be the case in maturity and aging (Pepe et al., 2017).

Microglia is an obvious phagocytic mediator in the CNS for maintaining normal brain microenvironment or CNS organization to manage difficult neuropathological situations (Neumann et al., 2008; Sierra et al., 2013). Most of the studies were concentrated to find the role of phagocytic microglia in various neuropathogenesis until recently. But now its role in neuronal tissue and circuitry development are gaining attention with ‘phagoptosis’ and ‘pruning’ (Vilalta & Brown, 2018). Microphotographs depicting microglia of late embryonic and matured age groups were the most efficient contenders of phagocytic behavior, which were also supported by FACS readings (Figure 4). It is well understood that microglia produce ROS in neuropathogenic inflammation due to degenerative damage or other stresses (Sierra et al., 2014; von Bernhardi et al., 2015). However, microglial ROS production when estimated through age continuum showed serious deviation with maturity. The observed natural trend of remarkable many folds increase of ROS production in matured microglia may otherwise be one of the important causative factors of neuronal damage in maturation and aging.

Present study was aimed to decipher modifications of microglial vital functions through development and maturity and to identify their most stable and adaptive age‐matched population which may serve as a suitable ex situ model for more detailed study on microglial cellular biology. Therefore, most of our studies were done on isolated microglial cells from brain parenchyma. Hence, simultaneous in situ observations were done to find the parity between their in situ states with ex situ behavioral manifestation. That, in turn, provided us clues to find connections and explanations for our observations and reduce artifactual redundancy. Increased MHC class II+ expressive microglia in mature adult stages with amoeboid transformation of microglia in that stage of maturity indicated higher phagocytic potential which was observed in our ex situ observation (Figures 4 and 5). Such transformation could be supported with the concept of ‘reactive microgliosis’ and high ROS production in aging microglia, and produced a probable cause of instability in primary culture (Figure 2) (Rivest, 2009; Santiago et al., 2017; Streit et al., 1999). In contrary, ramifications with fine branching were found in young adult brain which were previously described as ‘resting’ but ‘surveilling’ microglia where we observed lower phagocytic potential with less ROS production (Nimmerjahn et al., 2005; Streit et al., 1999). Such microglia with finely branched projections, we predicted, were nearly impossible to be retrieved during isolation and hence susceptible to damage, which might be a cause for lower stability and survival of them in primary culture (Figure 2). However, neonatal and late embryonic microglia with their rounded but smaller morphological appearance in situ and less ROS productivity, higher proliferative potential ex situ were observed as stable microglial population is coherence with their properties.

Microglia, as an essential responder of brain microenvironment, are highly alert to survey the surroundings and modulate its activities accordingly (Nimmerjahn et al., 2005; Walker & Yirmiya, 2016). To operate with such sensitivity, they express large number of receptors and act as a source or sink of a plethora of soluble factors, cytokines, and chemokines (Hanisch, 2002). We hypothesized that such response behavior is changing with the developing and maturing ages. This was primarily observed with differential responses of sustenance and propagation in the primary cultures of microglia from different age groups (Figure 2). Our investigation on microglia of different ages with selected cytokines, namely, GMCSF, MCSF, IL‐4, IL‐6, and IFNγ, which were already been reported to be involved in microglial differentiation (Ghosh, 2010; Hanisch, 2002; Kim & Nagai, 2010), showed varied degrees of alterations in cellular morphology, viability and adaptability (Figure 7 and Table 2) (also seen in Figures B–E in Supporting Information). These study designs were aimed to select the optimal cytokine dosing and responsive microglial age group that could be utilized in in vitro conditions to develop a model culture system. Relied on the external morphology of the cells with maximal morphometric responsiveness as a primary indicator with a large combination of cytokines and their concentrations and age groups forming 80 options altogether in our study; the neonatal infants were favored for their maximal sensitivity for cytokine induction (Figure 7). Therefore, it was found that microglia from neonatal rats were very stable in primary culture and capable to respond and adapt much efficiently for longer in vitro than others in age continuum. Hence, they may be the potent candidate for studying different phenomena of the cell in variably conditioned ex situ environment. The less effectiveness in developing an ex situ platform for adult microglia, in contrary, had been an important finding which can be correlated with intrinsic cellular properties showing reduced proliferative potential and remarkable hike in ROS production leading to cellular stress in microglia with maturation (Figures 3 and 4). This is directly correlated to the cytokine induced polarization state of the microglial population depending on IL‐1β, IFN‐γ, TNFα, IL‐6 mediated ‘classical activation’ pathway which found more prevalent in aging with elevated oxidative stress from NADPH oxidase activated hydroxyl radicles, superoxides generation and NO production in various conditions (Czeh et al., 2011; Kim & Nagai, 2010; Pathipati & Ferriero, 2017). Establishment of persistent microglial cell lines mostly from perinatal subjects, including a recent attempt of primary culture of adult microglia from a mouse actually taking sub‐adult microglia along with other current studies on transcriptome data of the cell, jointly supports our notion of higher consistency and adaptability of microglia derived from very early life stage of rodent models with their suitability for primary culture as found in the present study (Galatro et al., 2017; Singh et al., 2014; Timmerman et al., 2018).

## 5 CONCLUSION

Therefore, microglial morpho‐functional behavior throughout its ontogeny in the normal rodent brain showed us that their basic cellular properties have a specific pattern from perinatal stage to maturity. As we observed, in late embryonic and neonatal stages, microglia are not much dependent on ROS, which might be related with their functional responsibility of pruning and shaping CNS circuitry exerting minimal stress and now it has been supported with a current finding showing that they are using filopodial nibbling or ‘trogocytosis’ expressing PSD95 related to non‐phagocytic membrane exchange (Weinhard et al., 2018). Intermediate young adult microglia settling in the CNS are tranquil in phagocytic behavior with low ROS production, but keen for surveillance and maintenance. Whereas microglia from matured adult rats tending toward aging brain showed high phagocytic vigor with dependence on cellular ROS and having lower proliferative potential. Microglial damaging behavior with age and in disease are the derivatives of their inherent behavioral changes in the normally maturing and aging brains with their polarization. Furthermore, the observed pattern of the basic cellular properties in the maturation continuum indicates the primary reasons behind the failure to extend adult cells ex situ for a longer period. In situ observations also provided explainable coherence with the documented patterns of cellular behavior of microglia through developmental and maturation continuum. As cytokine response behavior demonstrated better efficiency in neonatal infant microglia to change morphologically with cellular adaptability and behavior, they meet the requirements to perform as an effective ex situ model in rodents. By documenting changing microglial behavior through ontogeny, the present study has formulated a rapid isolation and culture of microglia, identifying its best age‐matched responder group for working further on microglial biology. This study most importantly deciphered the shifting cellular properties of microglia with age, their differentiation toward the ROS dependent phagocytic mode from perinatal to matured stage only and found the best responding age for developing effective ex situ model for microglia.

## AUTHOR CONTRIBUTIONS

Payel Ghosh performed the majority of experiments of this work with Anirban Ghosh and helped him to arrange the data; Ishani Deb helped in maintaining the rodent model and measuring oxidative burst of the cells, Sandip Bandyopadhyay helped in some technical aspects and data interpretation. Anirban Ghosh conceptualized, designed, supervised, and interpreted the experiments with acquiring grants and has written and revised the manuscript.

## Supporting information

Supporting Information

## Data Availability

All data are represented in the manuscript and if required further, will be provided by the corresponding author.
