# Supplementary material for: Morpho‐functional variation and response pattern of microglia through rodent ontogeny showing infant microglia as stable and adaptive than matured
Source: Brain Behav. 2021 Aug 6;11(8):e2315. doi: 10.1002/brb3.2315 (PMC8413723; doi:10.1002/brb3.2315)
Supplement: Supplementary file 1 — Supporting Information [file BRB3-11-e2315-s001.pdf]

Statements for Supplementary Figures:

As per reviewer's suggestion, this section of Figures are presented in the Supplementary Section where Figure A-E have been provided with the graphical representation of morphological changes in microglia with the effect of cytokines. The results from these figures have been summarized in Table 2 in the article text. The figures as originally inserted from Sigma Plot software output, their quality and legend sizes are now beyond the authors' editable limits, hence, compromised. However, we used the secondary axis layer to clearly mention the parameters of the graphs represented on the morphometric data.

Supplementary Figures: Effect of selected cytokines at different concentrations on morphological parameters of microglia primary culture

For Review Only

Measurement of cellular morphology of microglia in culture in the conditioned media by MCSF, GMCSF, IL-4, IL-6 and IFNγ respectively in selected concentrations of 1ng/ml, 3ng/ml, 9ng/ml and 50ng/ml. The **Fig-A** shows four representative photomicrographs of conditioned microglia as captured and measured by phase-contrast microscopy for cell area, length and projection length by using NIS-BR software (see text). Depending on the measurements, graphical representations of the cellular parameters in box plots with median values and deviations were done in **Fig-B, C, D** and **E** each depicting an age group as ED18, D5, D45 and D240 respectively showing cytokines in rows and parameters in columns. The box plots are inserted as derived in Sigma Plot 13.0 software. Box plots are showing the differences of responses for cytokines in varied concentrations from where best doses were selected (see text).

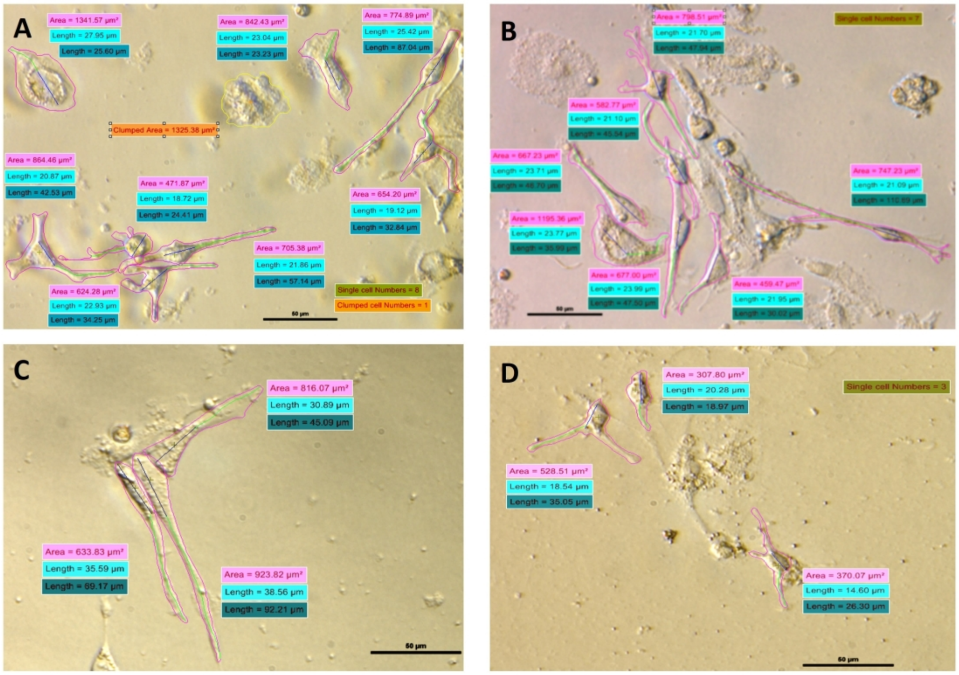

A

Representative micrographs of cellular morphometry: (A) ED 18, GMCSF, 3ng/ml; **(B)** D5, GMCSF, 1ng/ml; **(C)** D5, IL-6, 9ng/ml; **(D)** D45, IFNγ, 9ng/ml.

254x203mm (300 x 300 DPI)

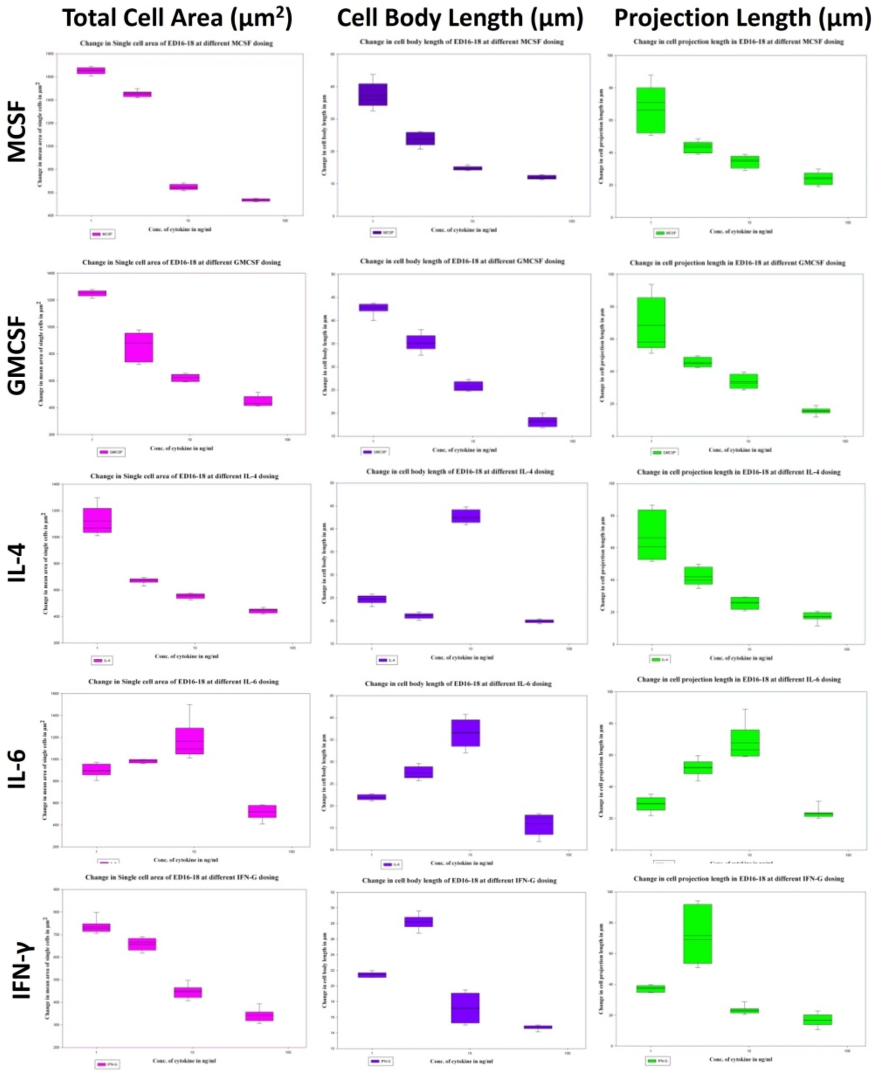

Effect of selected cytokines at different conc. on morphology of cultured microglia isolated from rat brain of embryonic day 18 [cytokine conc. 0-100ng/ml in log scale through X axis]

B

266x342mm (300 x 300 DPI)

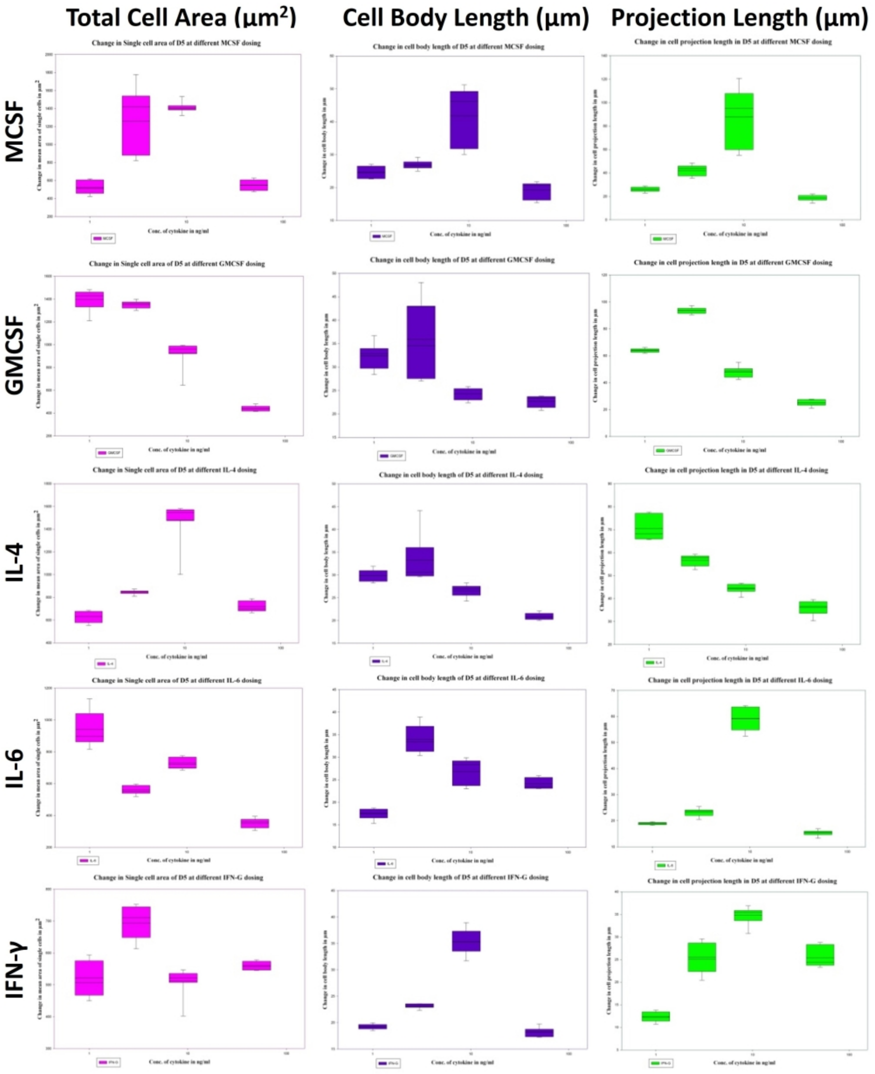

C Effect of selected cytokines at different conc. on morphology of cultured microglia isolated from rat brain of day 5 (neonates) [cytokine conc. 0-100ng/ml in log scale through X axis]

266x342mm (300 x 300 DPI)

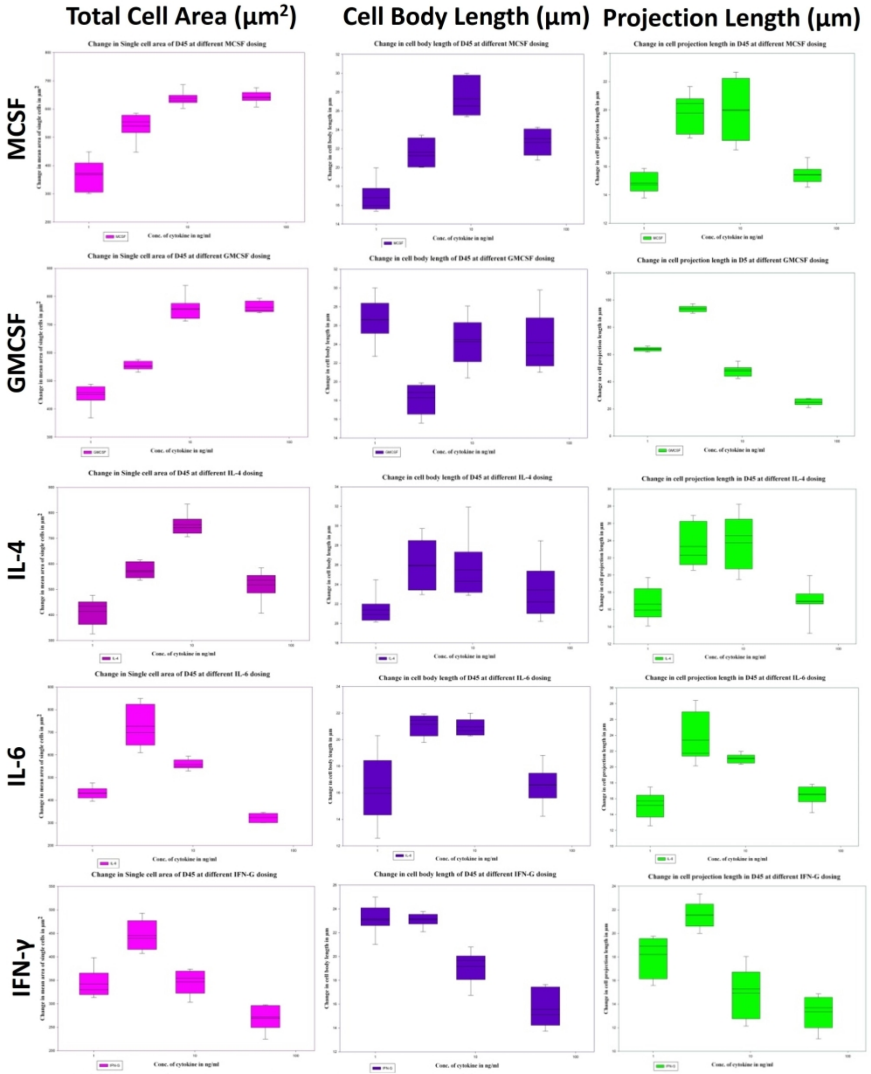

D Effect of selected cytokines at different conc. on morphology of cultured microglia isolated from rat brain of day 45 (young adult) [cytokine conc. 0-100ng/ml in log scale through X axis]

266x342mm (300 x 300 DPI)

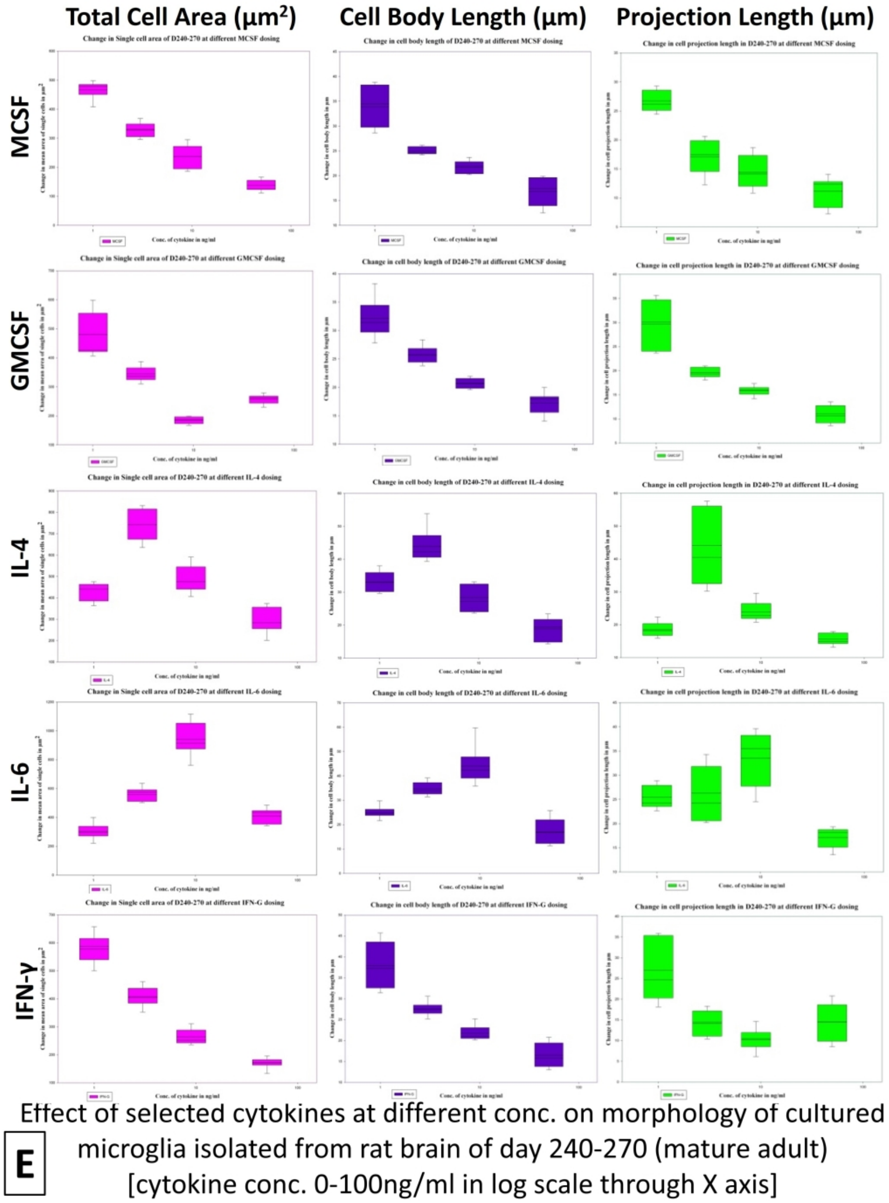

Effect of selected cytokines at different conc. on morphology of cultured microglia isolated from rat brain of day 240-270 (mature adult) [cytokine conc. 0-100ng/ml in log scale through X axis]

266x342mm (300 x 300 DPI)
